# Clinical effectiveness of restorative materials for the restoration of carious lesions in pulp treated primary teeth: a systematic review

**DOI:** 10.1007/s40368-022-00744-4

**Published:** 2022-09-03

**Authors:** S. Amend, C. Boutsiouki, K. Bekes, D. Kloukos, S. Gizani, N. N. Lygidakis, R. Frankenberger, N. Krämer

**Affiliations:** 1 Department of Paediatric Dentistry, Medical Centre for Dentistry, University Medical Centre Giessen and Marburg (Campus Giessen), Justus-Liebig-University Giessen, Schlangenzahl 14, 35392 Giessen, Germany; 2 Department of Paediatric Dentistry, University Clinic of Dentistry, Medical University Vienna, Sensengasse 2a, 1090 Vienna, Austria; 3 Department of Orthodontics and Dentofacial Orthopedics, University of Bern, Freiburgstrasse 7, 3010 Bern, Switzerland; 4 Department of Paediatric Dentistry, Athens School of Dentistry, National and Kapodistrian University of Athens, 2 Thivon Str, 115 27 Goudi, Athens, Greece; 5 Lygidakis Dental Clinic (Private Dental Practice), 2 Papadiamantopoulou str. and Vasilissis Sofias Ave, 115 28 Athens, Greece; 6 Department of Operative Dentistry, Endodontics and Paediatric Dentistry, Medical Centre for Dentistry, University Medical Centre Giessen and Marburg (Campus Marburg), Philipps-University Marburg, Georg-Voigt-Str. 3, 35039 Marburg, Germany

**Keywords:** Primary teeth, Caries, Pulp therapy, Restorative materials, Clinical effectiveness, Systematic review

## Abstract

**Purpose:**

To systematically review the clinical performance of restorative materials after pulp therapy of carious primary teeth. It is part 2 of a systematic review on the clinical effectiveness of restorative materials for the management of carious primary teeth supporting the European Academy of Paediatric Dentistry (EAPD) guideline development.

**Methods:**

Four electronic databases were systematically searched up to December 28th, 2020. Randomised controlled clinical trials (RCTs) on restorative materials for the restoration of carious primary teeth after pulp therapy were included. Failure rate, annual failure rate (AFR) and reasons for failure were recorded. Studies were sorted by restorative materials. The Cochrane Risk of bias tool for randomised trials (RoB 2.0) was used for quality assessment.

**Results:**

After identification of 1685 articles and screening of 41 papers from EAPD review group 1, 5 RCTs were included. Restored primary molars with pulpotomy presented the following AFRs: composite resin (CR) 0%, preformed metal crowns (PMCs) 2.4–2.5%, resin-modified glass-ionomer cement combined with CR 3.8%, compomer 8.9%, and amalgam 14.3%. Maxillary primary incisors receiving pulpectomy exhibited AFRs of 0–2.3% for composite strip crowns (CSCs) depending on the post chosen. Reasons for failure were secondary caries, poor marginal adaptation, loss of retention and fracture of restoration. All studies were classified as high risk of bias. Meta-analyses were not feasible given the clinical/methodological heterogeneity amongst studies.

**Conclusion:**

Considering any limitations of this review, CR and PMCs can be recommended for primary molars after pulpotomy, and CSCs for primary incisors receiving pulpectomy. However, a need for further well-designed RCTs was observed.

**Supplementary Information:**

The online version contains supplementary material available at 10.1007/s40368-022-00744-4.

## Introduction

Despite measurable progress in caries prevention, caries remains an unsolved problem worldwide (Bagramian et al. 2009) and it is reportedly affecting 2.4 billion adults and over 600 million children globally (Kassebaum et al. 2015). In the field of paediatric dentistry, this is of extreme importance as in many cases, mostly due to several socioeconomic reasons, infants arrive too late at the dentist's office with their parents. Therefore, caries is already frequently associated with pulpitis, which when irreversible, leads to pulp treatment or even to premature extractions (Alsheneifi and Hughes 2001), let alone the cost of untreated dental caries which is estimated to exceed $532 Mio, with richer countries showing a significantly lower prevalence (Vernazza et al. 2021).

Clinicians usually use conventional restorative treatment to manage dental caries in healthy primary teeth, as well as after vital and non-vital pulp therapy. Common restorative materials include dental amalgam, composite resins, compomers, glass-ionomer cements or resin-modified glass-ionomer cements, or paediatric crowns. Until the Minamata Convention in 2017 (Minamata Convention on Mercury 2013), when a reduction in the use of dental amalgam was agreed, amalgam has been used in paediatric dentistry (Hse and Wei 1997; Dutta et al. 2001; Sengul and Gurbuz 2015). Adhesive restorations have largely replaced amalgam in primary dentition; though their application is technique-sensitive and time-consuming (van de Sande et al. 2013; Opdam et al. 2014; Laske et al. 2016). Since success of restorations depends on several factors apart from the material itself, such as rubber dam, operators' skills, patients' characteristics, compliance and age of the child (Demarco et al. 2012; Chisini et al. 2018), considerable amounts of restoration failures have been reported in literature, mostly due to secondary caries (Opdam et al. 2014; Laske et al. 2016). In cases of pulp treated primary teeth, restoration failure would soon be translated into failure of the pulp therapy due to bacterial leakage (Boutsiouki et al. 2018). Taken into account that preservation of primary teeth in the oral cavity until physiological exfoliation is important for biological, functional and aesthetic reasons, an effective restoration over a vital and non-vital pulp therapy should be sought.

However, available restorative materials for primary teeth are characterised by strengths and limitations: Amalgam (A) is not a technically sensitive material but its preparation design causes more substance loss, which is contrary to the modern minimally invasive approach in dentistry (Daou et al. 2009; Hilgert et al. 2014). Composite resins (CRs) are minimally invasive, and a successful adhesion can be obtained to primary teeth when used in combination with universal adhesives (Lenzi et al. 2017). However, their technique-sensitivity has to be taken into consideration, which may be influenced by the operators’ experience and by a contamination during the application (Casagrande et al. 2013; Cavalheiro et al. 2020). For glass-ionomer cements (GICs), their use as bulk-fill restorative materials is advantageous in paediatric dentistry. Again, a limiting factor is the need for a more invasive preparation, as GICs need a retentive cavity design and they are susceptible to bulk fracture given their compromised mechanical properties (Kilpatrick et al. 1995; Espelid et al. 1999; Krämer and Frankenberger 2001), especially when large lesions with minimum support from the remaining tooth structures need to be restored, such as in cases of endodontically treated teeth. Compared to conventional GIC, resin-modified glass-ionomer cements (RMGICs) are characterised by an improved flexural strength. The limited wear resistance appears to be acceptable for the treatment of primary teeth (Hübel and Mejare 2003; Kotsanos and Arizos 2011). Preformed metal crowns (PMCs) are mainly indicated for extended carious defects, post-endodontic restorations, and for the Hall-technique (Innes et al. 2015). Despite their overall good performance, their aesthetics are a concern for parents visiting dental practises nowadays (Hutcheson et al. 2012; Donly et al. 2020). Instead, zirconia paediatric crowns can offer an aesthetic alternative (Alrashdi et al. 2021).

According to the American Academy of Pediatric Dentistry, based on complaints, medical history, and clinical diagnosis the pulpal status is differentiated between normal pulp, reversible pulpitis, (a-)symptomatic irreversible pulpitis, and necrotic pulp. (Pulp Therapy for Primary and Immature Permanent Teeth. The Reference Manual of Pediatric Dentistry 2020). Pulp therapy is indicated when reversible pulpitis (vital pulp therapy), irreversible pulpitis, or pulp necrosis (non-vital pulp therapy) is being diagnosed (Pulp Therapy for Primary and Immature Permanent Teeth. The Reference Manual of Pediatric Dentistry 2020). The present systematic review examined clinical studies that included both of the aforementioned types of pulp therapy in primary teeth and was performed due to deep caries.

Therefore, the purpose of this study was to systematically review the clinical effectiveness and reasons for failure of different restorative materials including new biomaterials for restoration of primary teeth after vital or non-vital pulp therapy due to caries.

## Methods

### Protocol, registration and reporting items

The protocol was registered in PROSPERO international prospective register of systematic reviews hosted by the National Institute for Health Research (NIHR), University of York, UK, Centre for Reviews and Dissemination (CRD42020221944) prior to the beginning. The Preferred Reporting Items for Systematic Reviews and Meta-Analyses (PRISMA) were followed during the entire process of this systematic review (Page et al. 2021).

### PICO(S) scheme

#### Population

Children of any sex and age with carious primary teeth subjected to a restorative treatment approach after vital or non-vital pulp therapy.

#### Intervention

(i) Any technique/degree of carious tissue removal (selective vs. complete) combined with the same/different restorative material(s) placed in primary teeth (i.e. adhesive/compomer (C), adhesive/composite resin (CR), glass-ionomer cement (GIC), resin-modified glass-ionomer cement (RMGIC), metal-reinforced glass-ionomer cement (MRGIC), bio-active materials (BM), amalgam (A), preformed metal/zirconia/composite crowns). (ii) The same approach for carious tissue removal in combination with any type of restorative material placed as restoration in primary teeth.

#### Comparison(s)

Conventional restorative approach using any other technique/degree of carious tissue removal and/or any other type of restorative material to restore carious lesions in primary teeth.

#### Outcome

Primary outcomes were: (i) Treatment failure (i.e. modified USPHS criteria (Ryge and Snyder 1973; Roulet 1994; Krämer et al. 1999)) and (ii) restoration quality. (iii) To assess the failure of crowns, the following outcome criteria needed to be described: Modified USPHS criteria for crowns (Alaki et al. 2020) or outcome criteria like success/major failure/minor failure (Santamaria et al. 2017).

Secondary outcomes were: (i) Time until restoration failure/re-treatment, (ii) discomfort during restorative treatment or within 24 h after treatment, (iii) patient’s/carer’s perceptions of the restorative treatment, (iv) factors influencing the clinical effectiveness of the restorative treatment: Type of tooth, affected tooth surface(s), preoperative radiograph, caries lesion depth, extent of carious tissue removal, isolation technique, type of adhesive and restorative material. Follow-up: Follow-up periods of at least 12 months were accepted.

#### Study design

Randomised controlled clinical trials (RCTs).

### Inclusion and exclusion criteria

The following inclusion criteria were defined:

- Any study fulfilling PICO(S) with dentine caries in primary teeth requiring intervention.
- Studies with primary teeth treated by vital (indirect pulp treatment, direct pulp capping, pulpotomy) or non-vital pulp therapy (pulpectomy).
- Teeth needed to be pulp treated and without any signs of symptoms or pathologies (i.e. pain, infection, swelling, furcal/periapical inflammation).
- Follow-up period of minimum 12 months and at least 40 restorations per group (Chisini et al. 2018).

The exclusion criteria were:

- Any type of study not fulfilling the inclusion criteria and not within the scope of this systematic review was excluded.
- Study designs other than RCTs.
- Studies with restorations conducted in permanent teeth.
- Studies with a drop-out rate > 30% (Tedesco et al. 2017).

### Search strategy

One experienced researcher (DK) developed the search strategies and searched the following electronic databases up to December 28th, 2020: MEDLINE/PubMed, EMBASE (via Ovid), Cochrane Library, and LILACS. The search strategy was appropriately adapted to the specific requirements (controlled vocabulary, syntax rules) of each electronic database. Appendix 1 shows the search strategies of all electronic databases. Restrictions were neither applied to the language nor to the publication time. Manual search was carried out to find further relevant studies. The reference lists of all included studies and of published systematic reviews on restorative treatment in primary teeth were hand searched by two authors (NNL, SA) for further eligible RCTs.

### Study selection

Study selection was conducted independently and in duplicate by two authors (CB, SA). Titles and abstracts of selected studies were screened according to the inclusion criteria by using Rayyan QCRI application for the initial filtering (Ouzzani et al. 2016). Full-texts of publications of possibly eligible studies were read and the relevance to the scope of this systematic review was judged. In case a study was published in several reports, the most recently published report showing the relevant outcomes for this systematic review was considered. Disagreements in any stage between the two authors performing the study selection were resolved by consensus-based discussion and by consultation of a third author (DK). The authors were not blinded to the study authors’ identities, the study institutions, and the outcomes of the RCTs. The decisions made during the study selection procedure were kept in record form.

### Data collection

Two authors (CB, SA) extracted the data from the ful-text reports of finally included RCTs independently. A calibration training was performed using 10 studies for data extraction and risk of bias assessment to compare the authors’ assessments and discuss the results with an experienced researcher in this field (DK). The full methodology is in analogy to a previous systematic review (Amend et al. 2022). In brief, reported failure rates and annual failure rates (AFRs) were extracted. In case survival rates of restorations were estimated by Kaplan–Meier statistics, failure rates were computed based on the outcome of the survival analysis. Moreover, the main reasons for restoration failure were recorded.

At first, sound primary teeth without any pulp treatment were included in part 1 of the systematic review. In a second step, the review question was extended to primary teeth treated by vital or non-vital pulp therapy: what is the clinical effectiveness of restorative materials including new biomaterials used for the restoration of carious primary teeth after vital or non-vital pulp therapy? In an attempt to answer this question, all included RCTs from the EAPD review group 1 were screened for eligibility (Stratigaki et al. 2022). The strict eligibility criteria of this systematic review and meta-analysis comprised the treatment of vital, symptomless primary posterior teeth with deep carious lesions by vital or non-vital pulp therapy under (local or general) anaesthesia and aseptic conditions with a follow-up of at least 24 months (Stratigaki et al. 2022).

### Calculation of failure rates

For calculation of failure rates, reported evaluation criteria (Ryge and Snyder 1973; Roulet 1994; Krämer et al. 1999; Hickel et al. 2007; Frencken 2009) were transferred into dichotomous outcomes (acceptable/unacceptable; Table 1) (Dias et al. 2018).

**Table 1:** Evaluation criteria of the included RCTs divided into dichotomous outcome data modified according to Dias et al. (2018)

Evaluation criteria and judgement		Parameters for failure assessment			
		Secondary caries	Marginal integrity	Fractures	Loss of retention
Modified USPHS criteria	Acceptable	Alpha, Bravo	Alpha, Bravo	Alpha, Bravo	Alpha, Bravo
	Unacceptable	Charlie, Delta	Charlie, Delta	Charlie, Delta	Charlie, Delta
FDI criteria	Acceptable	1, 2, 3	1, 2, 3	1, 2, 3	1, 2, 3
	Unacceptable	4, 5	4, 5	4, 5	4, 5
ART criteria	Acceptable	0, 1	0, 1	0, 1	0, 1
	Unacceptable	C	2, 5	3, 4	6, 7

According to Opdam et al. (2014), the AFR was calculated using the following formula (Opdam et al. 2014):

$$\left( {1 - y} \right)^{z} = \left( {1 - x} \right)$$

*x* = total failure rate at ‘*z*’ years

*y* = mean AFR

### Quality assessment of the included studies

The methodological quality of the selected studies was assessed independently by two authors (CB, SA), who were not blinded to study authors’ identities, study institutions and journals. The Cochrane Risk of Bias 2 (RoB 2) tool for randomized trials was used for quality assessment (Sterne et al. 2019). In addition, information provided in chapter 8 of the Cochrane Handbook for Systematic Reviews of Interventions were regarded (Higgins et al. 2019). Disagreements between the authors were resolved by consensus-based discussion and by consultation of a third author (DK).

### Data analysis

If included RCTs presented related comparisons and identical outcomes, meta-analyses were planned to be implemented. The substantial clinical/methodological heterogeneity among included primary studies did not allow for the conduction of meta-analyses.

## Results

### Selection of studies

**Fig. 1:**
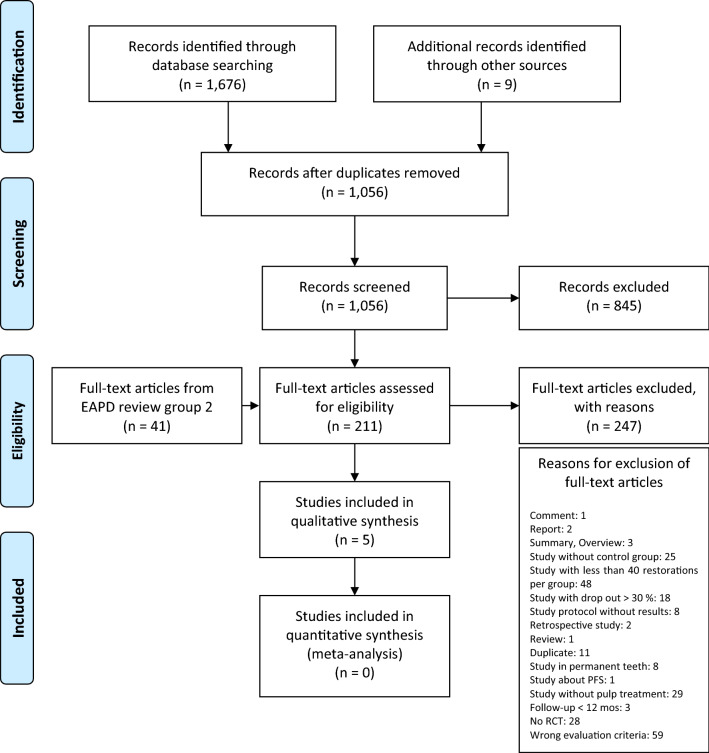
PRISMA flow diagram presenting the study selection process, the numbers of studies with vital pulp therapy or non-vital pulp therapy identified, eligible, and included in the systematic review. Modified according to: Moher D, Liberati A, Tetzlaff J, Altman DG, The PRISMA Group (2009). Preferred Reporting items for Systematic Reviews and Meta-Analyses: The PRISMA Statement. PLoS Med 6(7):e1000097. Doi:10.1371/journal.pmed1000097

Based on the selection criteria, 1676 articles were identified through database screening and 9 additional papers were retrieved through other sources (screening of reference lists, hand search). Among these records, 620 duplicates were removed. Another 845 records were excluded because the title and/or the abstract did not fulfil the inclusion criteria. A number of 211 full-text articles and 41 additional full-text articles from EAPD review group 1 were assessed for eligibility. The reasons for exclusion of 247 full-text articles are presented in Fig. 1. Thus, five articles remained for qualitative synthesis and no record was included in the quantitative analysis, due to the high risk of bias.

### Characteristics of included studies

Five RCTs assessing restoration failure in primary teeth with vital pulp therapy or non-vital therapy were included (Table 2). All studies used a split-mouth design and were published between 2006 and 2020. Sample size calculation was reported in two studies (Atieh 2008; Liberman et al. 2020). None of the studies obtained information about funding.

**Table 2:** RCTs with at least one-year follow-up evaluating restorations on primary teeth: the systematic review results for restorations after vital pulp therapy and non-vital pulp therapy

Author, year	Aim Study design Clinical setting (No of operators/evaluators) Funding (Yes (Source)/No) Country	Follow-up (years)	Inclusion criteria (according to the authors)	Included participants in total [age (years)] Gender (male:female)	Restoration at baseline/Last follow-up per group	Restorative material (material name)	Restoration class Isolation	Evaluation criteria	Failure rate/Annual failure rate/Annual failure rate according to Opdam et al., (2014)	Reasons for failure Secondary caries (SC) Marginal adaptation (MA) Fractures (F) Loss of Retention (LR) Radiographic pathology (RP)
Atieh (2008)	PMCs vs RMGIC + CR RCT, split-mouth General dental practise (1/1) NR Saudi Arabia	2	Healthy children No systemic disease No developmental disturbances of teeth/jaws Acceptable oral hygiene (plaque index score ≤ 20%) Behavioural rating score 3–4 (Frankl scale) At least 1 restorable carious primary molar with exposed vital pulp	87 (4–7) 41:46	PMC: 80/68 RMGIC + CR: 80/65	PMC (3 M) RMGIC + CR (Vitremer + Filtek Z250)	Primary molars NR Pulpotomy Rubber dam	Modified USPHS, Gingival health	PMC: 5%^a^/2.5%/2.5%^c^ RMGIC + CR: 7.5%^a^/3.8%/3.8%^c^	PMC: SC: 1%^a^ MA: 3%^a^ F: NR LR: 5%^a^ RP: NR RMGIC + CR: SC: 4%^a^ MA: 3%^a^ F: 3%^a^ LR: 1%^a^ RP: NR
Cehreli et al. (2006)	C vs CR RCT, split-mouth NR (2/2) NR Turkey	2	At least 2 primary molars with vital carious exposure during caries removal Bleeding from exposed pulp as expected from a vital pulp Remaining tooth tissue restorable with an occlusal or two-surface occluso-proximal restoration following complete caries removal	84 (6–10) NR	C: 100/72 CR: 100/80	C (Dyract) CR (TPH)	Primary molars Class I, II Pulpotomy Cotton rolls	Modified USPHS	C: 17%^a^/8.5%/8.9% CR: 0%^a^/0%/0%	C: SC: 0%^a^ MA: 0%^a^ F: 0%^a^ LR: NR RP: 17% CR: SC: 0%^a^ MA: 0%^a^ F: 0%^a^ LR: NR RP: 2%
Eshghi et al. (2013)	CP vs FP vs RMP RCT, split-mouth University (1/2) NR Iran	1	ECC involving 3/4 of the crown Sound root Sufficient amount of root structure present on radiographs (≤ 1/3 external root resorption compared with adjacent teeth)	54 (2–4) NR	CP: 53/43 FP: 54/45 RMP: 54/48	CP (Amelogen), CSC (Amelogen) FP (Biscem), CSC (Amelogen) RMP (NR), CSC (Amelogen)	Maxillary primary incisors NR Pulpectomy Cotton rolls	FDI	CP: 2.3%/2.3%/2.3% FP: 2.2%/2.2%/2.2% RMP: 0%/0%/0%	CP: SC: NR MA: 2% F: 0% LR: NR RP: NR FP: SC: NR MA: 16% F: 7% LR: NR RP: NR RMP: SC: NR MA: 10% F: 6% LR: NR RP: NR
Liberman et al. (2020) Franzon et al. (2015)	Complete vs selective caries removal, CR RCT, split-mouth University (3/1) Nil Brazil	2–3	Good general health Primary teeth with deep caries lesions in dentine in 1–2 surfaces Radiographically located in the inner quarter of dentine No sensitivity, spontaneous pain, swelling, fistula, mobility incompatible with the root resorption stage No periapical/interradicular radiolucency, or other radiographic signs indicative of pulp necrosis	48 (3–8) 25:23 (children attending follow-up, not all randomised)	CCR: 57/55 SCR: 67/65	CCR (Filtek Z350) SCR (Filtek Z350)	Primary molars Class I, II Pulpotomy^f^ Rubber dam	Modified USPHS, Gingival bleeding index	CCR: 19%^a^/6.7%/6.8^c^ SCR: 43%^a^/17.3%/17.1^c^	CCR at 24 mos: SC: 9.4% MA: 13.2% F: 7.5% LR: NR RP: NR SCR at 24 mos: SC: 21.2% MA: 25.8% F: 17.7% LR: NR RP: NR
Sonmez and Duruturk (2010)	A vs PMC RCT, split-mouth University (NR/2) NR Turkey	1	Deep carious lesions (radiographically in proximity to the pulp) Exposure of vital pulp during caries excavation Root resorption of < 1/3 of total root length Possibility of restoration following pulpotomy No symptoms of advanced pulpal inflammation No clinical symptoms suggesting a non-vital tooth No radiographically demonstrable pathology Cessation of haemorrhaging of the amputated pulp stump within 5 min	83 (4–9) 44:39 (children attending follow-up, not all randomised)	A: 70/70 PMC: 84/84	A (SDI) PMC (3 M)	Primary molars NR Pulpotomy Cotton rolls	Clinical, radiographic success	A: 14.3%/14.3%/14.3% PMC: 2.4%/2.4%/2.4%	A: SC: NR MA: NR F: NR LR: NR RP: NR PMC: SC: NR MA: NR F: NR LR: NR RP: NR

*RCT* randomised controlled clinical trial, *A* amalgam, *C* compomer, *CCR* complete caries removal, *CP* composite post, *CR* composite resin, *CSC* composite strip crown, *ECC* early childhood caries, *ER* etch-and-rinse, *F* fracture, *FP* fibre post, *GIC* glass-ionomer cement, *LR* loss of retention, *MA* marginal adaptation, *NR* not reported, *RMGIC* resin-modified glass-ionomer cement, *RMP* reversed metal post,  *RP* radiographic pathology, *SC* secondary caries, *SCR* selective caries removal, *SE* self-etch, *PMC* preformed metal crown

^a^According to the authors (information written by authors of the trial in the paper)

^c^Results based on Kaplan–Meier statistics

^f^In cases of pulp exposure

The studies were carried out in Saudi Arabia (Atieh 2008), Turkey (Cehreli et al. 2006; Sonmez and Duruturk 2010), Iran (Eshghi et al. 2013), and Brazil (Liberman et al. 2020). Three studies were conducted at university (Sonmez and Duruturk 2010; Eshghi et al. 2013; Liberman et al. 2020), one in a general dental practise (Atieh 2008), and in one study information about the setting were not obtained (Cehreli et al. 2006).

A sum of 808 teeth were randomly selected for treatment in 364 children aged 2–10 years. The gender ratio was reported in three studies. In these studies, 47.1–54.9% of participants were male (Atieh 2008; Sonmez and Duruturk 2010; Liberman et al. 2020).

A number of 695 restorations in primary teeth were evaluated after a mean follow-up of 21.6 months (± 10 months). Except for one study including severely decayed maxillary incisors (Eshghi et al. 2013), primary molars were chosen for restorative treatment (Cehreli et al. 2006; Atieh 2008; Sonmez and Duruturk 2010; Liberman et al. 2020). The cavity class was not reported in three studies (Atieh 2008; Sonmez and Duruturk 2010; Eshghi et al. 2013). In the remaining two studies, Class-I and Class-II cavities were prepared (Cehreli et al. 2006; Liberman et al. 2020). The pulp therapy comprised pulpotomy in four studies (Cehreli et al. 2006; Atieh 2008; Sonmez and Duruturk 2010; Liberman et al. 2020) with one study only performing a pulpotomy in case of pulp exposure during caries removal. In the remaining study, teeth received pulpectomy prior to post insertion (Eshghi et al. 2013). Modified USPHS criteria (Cehreli et al. 2006; Atieh 2008; Liberman et al. 2020), FDI criteria (Eshghi et al. 2013), and own evaluation criteria (Sonmez and Duruturk 2010) were used to assess the clinical success.

One study, reported in several articles, evaluated the impact of the caries removal technique (selective vs complete) on the survival of Class-I and Class-II composite resin restorations in primary molars after 36 months (Franzon et al. 2014, 2015; Liberman et al. 2020). The authors included 48 of the participants in the final analysis. After caries removal, a calcium hydroxide cement was applied as lining. All teeth were restored using a 2-step etch-and-rinse adhesive and a composite resin. In this study, a pulpotomy with 15.5% ferric sulphate was solely performed in case of pulp exposure during caries removal. A previous publication presenting the 24 months results of the same study reported that one pulp was exposed during SCR. In contrast to this, pulp exposure occurred in 15 teeth of the CCR group (Franzon et al. 2015). The survival rate of all teeth with pulpotomy was 92% after 24 months (Franzon et al. 2015).

Three included studies investigated the clinical performance of restorations placed after pulpotomy in primary molars (Cehreli et al. 2006; Atieh 2008; Sonmez and Duruturk 2010). All pulpotomies were performed after administering local anaesthesia and under isolation with either rubber dam (Atieh 2008) or cotton rolls (Cehreli et al. 2006; Sonmez and Duruturk 2010). Full-strength formocresol (Cehreli et al. 2006), diluted formocresol (Atieh 2008), or calcium hydroxide (Sonmez and Duruturk 2010) were used during pulpotomy in primary molars. Zinc-oxide eugenol cement was applied to cover the radicular pulp stumps in the three studies (Cehreli et al. 2006; Atieh 2008; Sonmez and Duruturk 2010). Teeth receiving pulpotomy were restored with amalgam (Sonmez and Duruturk 2010), modified open-sandwich restorations (RMGIC + composite resin) (Atieh 2008), compomer (Cehreli et al. 2006), composite resin (Cehreli et al. 2006), or preformed metal crowns (Atieh 2008; Sonmez and Duruturk 2010). The adhesive system used was not reported for modified open-sandwich restorations (Atieh 2008). Prime&Bond NT was chosen for compomer (applied as 1-step self-etch product) and composite resin restorations (applied as 2-step etch-and-rinse product) (Cehreli et al. 2006).

One study evaluated the clinical performance of composite strip crowns filled with a microhybrid composite resin (Amelogen, Ultradent, South Jordan, Utah, USA) in combination with posts in maxillary primary incisors with pulpectomy (Eshghi et al. 2013).

No studies were found that reported on the use of restorative materials containing bio-active compounds for the restoration of carious primary teeth after vital or non-vital pulp therapy (Imazato et al. 2014).

### Quality assessment of the included studies

**Fig. 2:**
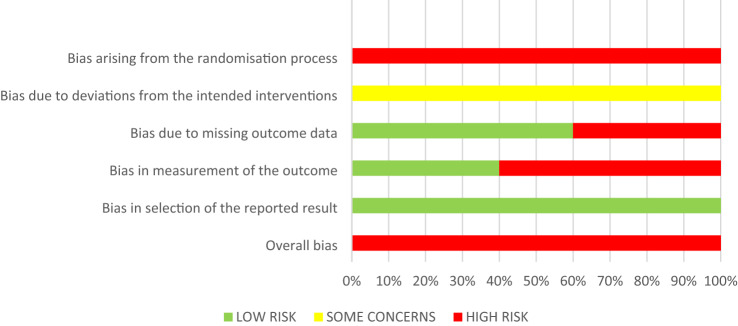
Summary of the risk of bias assessment

The five RCTs were assessed to be at high risk of bias. The main reasons for the high risk of bias of the included RCTs were attributed to randomisation sequence generation and allocation concealment (100% of the RCTs), missing outcome data (40%), and measurement of the outcome (60%, Table 3, Fig. 2).

**Table 3:**
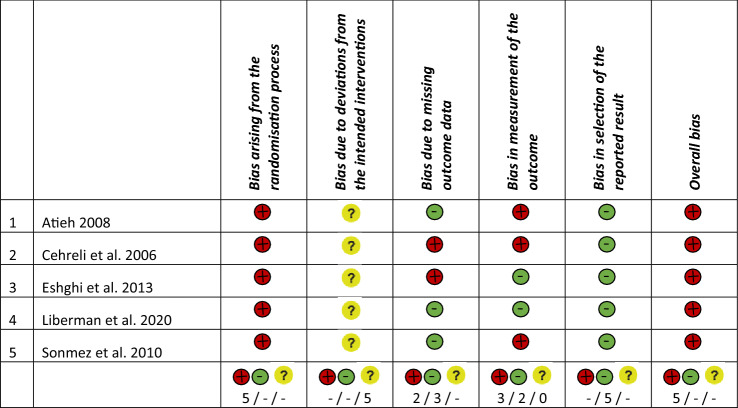
Risk of bias assessment of the included RCTs

#### Bias arising from the randomisation process

All five studies were rated as being at high risk of selection bias given an insufficient reporting, especially for the allocation sequence concealment. In three studies, allocation sequence generation was achieved by a computer-generated list (Atieh 2008) or by coin toss (Sonmez and Duruturk 2010; Liberman et al. 2020). Two studies did not provide information about the randomisation process (Cehreli et al. 2006; Eshghi et al. 2013). None of the five studies reported attempts to conceal the allocation sequence (Cehreli et al. 2006; Atieh 2008; Sonmez and Duruturk 2010; Eshghi et al. 2013; Liberman et al. 2020).

#### Bias due to deviations from the intended interventions

Some concerns prevailed considering the risk of performance bias among the included studies (Atieh 2008; Cehreli et al. 2006; Eshghi et al. 2013; Liberman et al. 2020; Sonmez and Duruturk 2010). The rationale was the same as already described in a previous systematic review (Amend et al. 2022); namely, the impossibility of blinding participants and trial personnel in case of using different restorative treatment approaches.

#### Bias due to missing outcome data

60% (*n* = 3) of the included studies (Atieh 2008; Sonmez and Duruturk 2010; Liberman et al. 2020) were rated as being of low risk of bias due to missing outcome data since the drop-out rate was below 10%. The other 40% of included studies had a high risk of attrition bias (drop-out rate > 10%) (Cehreli et al. 2006; Eshghi et al. 2013).

#### Bias in measurement of the outcome

Two studies used the same restorative material for all intervention groups allowing for a blinding of outcome assessors (Eshghi et al. 2013; Liberman et al. 2020). The remaining three studies were rated as being of high risk of bias. Among them, in one study the operator and the outcome assessor were the same person (Atieh 2008) and in the other two studies, restorative materials of different appearance were chosen to restore primary molars with pulpotomy (Cehreli et al. 2006; Sonmez and Duruturk 2010).

#### Bias in selection of the reported results

All included studies were at low risk of bias for selective reporting (100%, *n* = 5) (Cehreli et al. 2006; Atieh 2008; Sonmez and Duruturk 2010; Eshghi et al. 2013; Liberman et al. 2020).

### Reported outcomes for the restorative materials

#### Amalgam

In 1 study meeting the inclusion criteria, amalgam was chosen as restorative material for the assessment of its clinical effectiveness. The authors described that amalgam restorations placed after pulpotomy in primary molars presented an AFR of 14.3%.

The result for amalgam as restorative material for primary molars with pulpotomy is of low quality of evidence given the high risk of bias of this study.

#### Compomer and composite resin

As far as adhesive dentistry in pulp treated primary teeth is concerned, compomer restorations were evaluated in one study (Cehreli et al. 2006) and composite resin restorations in three studies (Cehreli et al. 2006; Atieh 2008; Liberman et al. 2020), of which in one study modified open-sandwich restorations with RMGIC plus composite resin were described as restorative treatment approach (Atieh 2008). For primary molars with pulpotomy, the choice of modified open-sandwich restorations resulted in an AFR of 3.8% (Atieh 2008).

In the study by Cehreli et al. (2006), the AFRs of Class-I and Class-II restorations in primary molars with pulpotomy were 0% for composite resin and 8.9% for compomer. Besides the different restorative materials chosen, the bonding strategy differed as well. Although Prime&Bond NT was used as adhesive system in both groups, it was either applied as 1-step self-etch product (compomer restorations) or as 2-step etch-and-rinse product (composite resin restorations) (Cehreli et al. 2006).

One study evaluated the impact of the caries removal technique on the survival of composite resin restorations. After caries removal, teeth either received an indirect pulp capping with calcium hydroxide or a pulpotomy in case of pulp exposure during caries removal. Selective caries removal (SCR) resulted in a higher annual failure rate (17.1%) of Class-I and Class-II composite resin restorations placed in combination with a 2-step etch-and-rinse adhesive than complete caries removal (CCR, 6.8%). Compared to complete caries removal, the probability of failure was 3.44 higher after selective caries removal. Moreover, the Class-II restorations and an impaired oral hygiene were mentioned as risk factors for failure (Liberman et al. 2020).

The findings for adhesively bonded restorations with compomer or composite resin after different types of pulp treatment (indirect pulp treatment, pulpotomy) are based on studies with a high risk of bias indicating a low quality of evidence.

#### Crowns

For severely decayed maxillary primary incisors, the AFRs of composite strip crowns placed in combination with composite posts, fibre posts, or reverse metal posts after pulpectomy ranged from 0 to 2.3% (Eshghi et al. 2013). The microhybrid composite resin Amelogen (Ultradent) was used for the crown reconstruction in all groups (Eshghi et al. 2013).

Preformed metal crowns were used in 2 studies to restore primary molars after pulpotomy. The reported AFRs for preformed metal crowns were 2.4, and 2.5% respectively (Atieh 2008; Sonmez and Duruturk 2010).

Again, the evidence was of low quality for these results, as all included studies on the clinical effectiveness of crowns in pulp treated primary teeth were at high risk of bias.

### Quantitative synthesis of the included studies

A well-grounded interpretation of the results by means of pooled estimates was not feasible given the high methodological heterogeneity (i.e. different interventions, follow-up periods, outcome criteria) of included studies. Moreover, the overall high risk of bias of all five included studies did not allow for a quantitative synthesis of results.

## Discussion

The aim of this systematic review was to evaluate the quality of the evidence of published RCTs on contemporary restorative treatment approaches in pulp treated primary teeth. Several studies investigated vital and non-vital pulp therapy approaches for primary teeth with deep caries ranging from indirect pulp treatment, direct pulp capping, to pulpotomy and pulpectomy (Coll et al. 2017; Tedesco et al. 2020). However, since the objective of those studies was primarily to assess the endodontic outcome, little information on restorative materials and techniques was given.

The majority of trials assessed clinical and radiographic success of vital and non-vital pulp therapy per se. Clinical success was defined as absence of pain, tenderness to percussion, pathological mobility, and inflammation. Teeth were deemed radiographically successful if they were free of external/internal root resorption, and furcal/periapical radiolucency (Demir and Cehreli 2007; Büyükgüral and Cehreli 2008; Coll et al. 2017; Celik et al. 2019). However, the outcome of the restorative treatment following the vital and non-vital pulp therapy was reported only in a few trials. Among these trials, some assessed marginal integrity of restorations in particular to evaluate if clinically visible microleakage was associated with clinical and/or radiographic failure of the vital pulp therapy (Demir and Cehreli 2007; Büyükgüral and Cehreli 2008; Celik et al. 2013).

Büyükgüral and Cehreli (2008) found no correlation between the deterioration of compomer and amalgam restorations’ marginal integrity and the clinical outcome of indirect pulp treatment in primary molars after 24 months (Büyükgüral and Cehreli 2008). This finding was confirmed by Demir and Cehreli (2007) for primary molars treated with adhesive pulp capping and Class-I restorations with either amalgam or compomer after haemostasis with 1.25% sodium hypochlorite. The outcome of the marginal quality assessment was not correlated to the clinical and/or radiographic failure observed during a 24 months follow-up (Demir and Cehreli 2007). Celik et al. (2013) confirmed this observation for primary molars with pulpotomy, which were restored with Class-I amalgam restorations during a follow-up of 24 months (Celik et al. 2013). Given the fact that in the trials mentioned above the restoration quality assessment was limited and did not cover all criteria of the USPHS rating system, these trials were not included in the present systematic review (Demir and Cehreli 2007; Büyükgüral and Cehreli 2008; Celik et al. 2019).

The five included RCTs investigated a broad spectrum of restorative treatment approaches after pulpotomy (with one trial performing this only in case of pulp exposure during caries removal) (Cehreli et al. 2006; Atieh 2008; Sonmez and Duruturk 2010; Liberman et al. 2020) or pulpectomy (Eshghi et al. 2013).

In primary molars with pulpotomy, composite resin restorations and preformed metal crowns achieved the lowest annual failure rates followed by open-sandwich restorations with RMGIC and composite resin (Cehreli et al. 2006; Atieh 2008; Sonmez and Duruturk 2010). Higher annual failure rates were observed for compomer and amalgam restorations (Cehreli et al. 2006; Sonmez and Duruturk 2010). For adhesive restorations, the adhesive approach should be taken into account as well. It was shown by Cehreli et al. (2006) that Class-I and Class-II compomer restorations had a higher risk of pulpotomy failure due to coronal microleakage compared to composite resin restorations. Apart from different restorative materials that were chosen in this study, the bonding differed as well. Whereas Prime&Bond NT was used as 1-step self-etch product in combination with compomer, it was applied as 2-step etch-and-rinse adhesive when composite resin was used (Cehreli et al. 2006). Besides the restorative material's characteristics, the bonding approach may have additionally provoked a failure in marginal adaptation leading to microleakage in the following. However, a systematic review and meta-analysis by Coll et al. (2017) revealed that the choice of the final restoration (PMC vs intracoronal restoration) did not significantly influence the success rates of vital pulp therapy at 24 months (82.4 vs 84.2%) (Coll et al. 2017).

Composite strip crowns placed in maxillary primary incisors with pulpectomy after post insertion exhibited the lowest annual failure rate when reversed metal posts were used. Slightly higher annual failure rates were observed for composite posts and fibre posts (Eshghi et al. 2013).

The five included RCTs of this systematic review were rated as being at “high risk of bias” (Table 3). Given the substantial heterogeneity (e.g. regarding study designs, chosen comparisons, selected outcome measures) combined with the overall high risk of bias of included studies, a meta-analysis was not feasible. Therefore, it was not possible to formulate specific recommendations for dental practise based on pooling effects from the available evidence.

The particular strength of this systematic review is that a broad spectrum of restorative materials for the restoration of carious primary teeth after vital or non-vital pulp therapy was included by applying strict eligibility criteria. The search strategy was neither restricted by language nor by publication year to reduce the possible risk of bias initiated by the literature search (Higgins et al. 2019). Additionally, only RCTs were included to minimise the risk of selection bias (Schwendicke et al. 2016). By focussing on the outcome of the restorative treatment after vital or non-vital pulp therapy an attempt was made to close the existing knowledge gap.

Likewise, the limitations of this systematic review need to be addressed. Although the original search strategy was broadly formulated there is the possibility that some studies were not retrieved, as specific search terms for vital and non-vital pulp therapy were not included. To overcome this shortcoming, the included RCTs on the management of deep carious lesions in primary teeth were additionally screened for eligibility (Stratigaki et al. 2022). Again, some studies may have been missed out given the strict eligibility criteria applied for the systematic review and meta-analysis (Stratigaki et al. 2022). By waiving to restrict the included studies based on the publication year, older versions of the restorative materials were not excluded. In general, there is the possibility that these materials present an inferior long-term performance compared to newer, modified products. This influence may be limited for the present systematic review, as the included studies were published between 2006 and 2020. The inconsistent reporting of teeth with physiological exfoliation, participants with loss to follow-up and censored data hampered the calculation of annual failure rates. All in all, the main limitations of this systematic review are the heterogeneity of study designs, the diversity of restorative materials under investigation in the included RCTs, and the high risk of bias of included studies, which impeded to draw recommendations for the best restorative approach in primary teeth after vital and non-vital pulp therapy. Especially after vital and non-vital pulp therapy, a tight coronal seal appears to be a fundamental prerequisite for long-term success. In accordance with the systematic review and network meta-analysis by Schwendicke et al. 2016, the results of the present systematic review need be interpreted cautiously since the majority of RCTs had a short-term follow up and a low quality of evidence (Schwendicke et al. 2016).

The results of the present systematic review highlighted a need for further well-designed RCTs on restorative treatment in primary teeth. To allow for a better comparability of results, standardised trial protocols should be implemented (Schwendicke et al. 2016) taking the following aspects into consideration: powered RCTs of parallel group design comparing restorative interventions are recommendable to overcome to limitations mentioned for studies in split-mouth design (Pozos-Guillen et al. 2017). The randomisation process and the allocation sequence concealment need to be selected thoroughly to prevent imbalances between intervention groups (Higgins and Thomas 2021). The age range of included participants should not be too wide as in older children the life expectancy of primary teeth is reduced due to the physiological root resorption and exfoliation. A description of the caries experience among the included participants, by using the dmf-t/DMF-T index for instance, helps to assess the caries risk, which has an impact on the interpretation of results. According to Opdam et al. (2014), a high caries risk is associated with an increased susceptibility for restoration failure (Opdam et al. 2014). Detailed descriptions of the interventions (availability of preoperative radiographs, assessment of carious lesion depth, administration of local anaesthesia, isolation technique, extent of carious tissue removal, restorative materials and application mode etc.) facilitate the comparisons between studies. Especially for adhesive restorations, the chosen adhesive protocol needs to be described in detail. The operator experience should be clearly stated as it was shown by Bücher et al. (2015) that the survival of restorations is influenced by the operator skills (Bücher et al. 2015). The implementation of internationally accepted outcome criteria, e.g. FDI criteria (Hickel et al. 2010) or modified USPHS criteria (Ryge and Snyder 1973), is required to achieve a higher standardisation permitting a comparison of results between the studies. Longer follow-ups are desirable to give a hint of a material’s long-term performance. For instance, it is more likely to detect secondary caries as reason for failure in studies with longer follow-up periods (Opdam et al. 2014; Schwendicke et al. 2016). In this respect, a precise report of the numbers of patients with loss to follow-up (including reasons for the withdrawal) and of the number of exfoliated teeth are of interest.

All in all, this systematic review confirmed the need for future RCTs evaluating restorative treatment approaches in primary teeth by adopting strict trial designs, by detailed reporting allowing for a better comparison of studies (Schwendicke et al. 2016), and by systematically assessing the restoration quality using internationally accepted criteria (i.e. FDI criteria, modified USPHS criteria).

## Conclusions

Considering any limitations of the present review, the following conclusions can be made:

- For the restoration of primary molars after pulpotomy, amalgam showed the highest AFR, followed by compomer, open-sandwich technique with RMGIC plus composite resin, PMCs, and composite resin at 12–24 months. For that reason, and also due to environmental concerns, amalgam is not recommended for use as a restorative material post-pulp treatment.
- The extent of caries removal and the adhesive strategy may influence the longevity of Class-I and Class-II composite resin restorations in primary molars after pulpotomy at 24–36 months.
- Primary molars receiving pulpotomy and PMCs exhibited a favourable retention rate at 12–24 months.
- For primary incisors with pulpectomy, composite strip crowns presented a low failure rate at 12 months.
- However, there is a need for further well-designed RCTs investigating the long-term success of the restorations after vital and non-vital pulp therapy to improve the quality of evidence for treatment recommendations.

## Supplementary Information

Below is the link to the electronic supplementary material.

Supplementary file1 (PDF 54 KB)
