# Supplementary material for: Clinical effectiveness of restorative materials for the restoration of carious lesions in pulp treated primary teeth: a systematic review
Source: Eur Arch Paediatr Dent. 2022 Sep 3;23(5):761–76. doi: 10.1007/s40368-022-00744-4 (PMC9637617; doi:10.1007/s40368-022-00744-4)
Supplement: Supplementary file 1 — Supplementary file1 (PDF 54 KB) [file 40368_2022_744_MOESM1_ESM.pdf]

Appendix 1. Search strategies of electronic databases

Nr.	Query	Filters	Results
**Medline/ Pubmed.** Clinical effectiveness of restorative materials including new biomaterials for the restoration of carious primary teeth.			
1	(primary[Title/Abstract] OR baby[Title/Abstract] OR deciduous[Title/Abstract] OR milk[Title/Abstract]) AND (tooth[Title/Abstract] OR teeth[Title/Abstract] OR dental[Title/Abstract] OR dentition[Title/Abstract])		29,204
2	(restorati*[Title/Abstract] OR crown[Title/Abstract] OR filling*[Title/Abstract]) AND (cari*[Title/Abstract] OR decay[Title/Abstract] OR cavities[Title/Abstract] OR dentine[Title/Abstract] OR lesion[Title/Abstract])		17,1
3	((restorati*[Title/Abstract] OR crown[Title/Abstract] OR filling*[Title/Abstract]) AND (cari*[Title/Abstract] OR decay[Title/Abstract] OR cavities[Title/Abstract] OR dentine[Title/Abstract] OR lesion[Title/Abstract])) AND ((primary[Title/Abstract] OR baby[Title/Abstract] OR deciduous[Title/Abstract] OR milk[Title/Abstract]) AND (tooth[Title/Abstract] OR teeth[Title/Abstract] OR dental[Title/Abstract] OR dentition[Title/Abstract]))		1,778
4	glass[Title/Abstract] OR polyalkenoate[Title/Abstract] OR ionomer[Title/Abstract] OR cement*[Title/Abstract] OR resin*[Title/Abstract] OR metal[Title/Abstract] OR composite*[Title/Abstract] OR amalgam[Title/Abstract] OR compomer*[Title/Abstract] OR Polyacid[Title/Abstract] OR biomaterial[Title/Abstract] OR bio-active[Title/Abstract]		673,865
5	(glass[Title/Abstract] OR polyalkenoate[Title/Abstract] OR ionomer[Title/Abstract] OR cement*[Title/Abstract] OR resin*[Title/Abstract] OR metal[Title/Abstract] OR composite*[Title/Abstract] OR amalgam[Title/Abstract] OR compomer*[Title/Abstract] OR Polyacid[Title/Abstract] OR biomaterial[Title/Abstract] OR bio-active[Title/Abstract]) AND (((restorati*[Title/Abstract] OR crown[Title/Abstract] OR filling*[Title/Abstract]) AND (cari*[Title/Abstract] OR decay[Title/Abstract] OR cavities[Title/Abstract] OR dentine[Title/Abstract] OR lesion[Title/Abstract])) AND ((primary[Title/Abstract] OR baby[Title/Abstract] OR deciduous[Title/Abstract] OR milk[Title/Abstract]) AND (tooth[Title/Abstract] OR teeth[Title/Abstract] OR dental[Title/Abstract] OR dentition[Title/Abstract])))		856
6	(glass[Title/Abstract] OR polyalkenoate[Title/Abstract] OR ionomer[Title/Abstract] OR cement*[Title/Abstract] OR resin*[Title/Abstract] OR metal[Title/Abstract] OR composite*[Title/Abstract] OR amalgam[Title/Abstract] OR compomer*[Title/Abstract] OR Polyacid[Title/Abstract] OR biomaterial[Title/Abstract] OR bio-active[Title/Abstract]) AND (((restorati*[Title/Abstract] OR crown[Title/Abstract] OR filling*[Title/Abstract]) AND (cari*[Title/Abstract] OR decay[Title/Abstract] OR cavities[Title/Abstract] OR dentine[Title/Abstract] OR lesion[Title/Abstract])) AND ((primary[Title/Abstract] OR baby[Title/Abstract] OR deciduous[Title/Abstract] OR milk[Title/Abstract]) AND (tooth[Title/Abstract] OR teeth[Title/Abstract] OR dental[Title/Abstract] OR dentition[Title/Abstract])))	Humans	689
7	(primary teeth[MeSH Terms]) AND (glass[Title/Abstract] OR polyalkenoate[Title/Abstract] OR ionomer[Title/Abstract] OR cement*[Title/Abstract] OR resin*[Title/Abstract] OR metal[Title/Abstract] OR composite*[Title/Abstract] OR amalgam[Title/Abstract] OR compomer*[Title/Abstract] OR Polyacid[Title/Abstract] OR biomaterial[Title/Abstract] OR bio-active[Title/Abstract])		1,49

**8**	(primary teeth[MeSH Terms]) AND (glass[Title/Abstract] OR polyalkenoate[Title/Abstract] OR ionomer[Title/Abstract] OR cement*[Title/Abstract] OR resin*[Title/Abstract] OR metal[Title/Abstract] OR composite*[Title/Abstract] OR amalgam[Title/Abstract] OR compomer*[Title/Abstract] OR Polyacid[Title/Abstract] OR biomaterial[Title/Abstract] OR bio-active[Title/Abstract])	Humans	1,417
**9**	((primary teeth[MeSH Terms]) AND (glass[Title/Abstract] OR polyalkenoate[Title/Abstract] OR ionomer[Title/Abstract] OR cement*[Title/Abstract] OR resin*[Title/Abstract] OR metal[Title/Abstract] OR composite*[Title/Abstract] OR amalgam[Title/Abstract] OR compomer*[Title/Abstract] OR Polyacid[Title/Abstract] OR biomaterial[Title/Abstract] OR bio-active[Title/Abstract]) AND (humans[Filter])) AND (((restorati*[Title/Abstract] OR crown[Title/Abstract] OR filling*[Title/Abstract]) AND (cari*[Title/Abstract] OR decay[Title/Abstract] OR cavities[Title/Abstract] OR dentine[Title/Abstract] OR lesion[Title/Abstract])) AND ((primary[Title/Abstract] OR baby[Title/Abstract] OR deciduous[Title/Abstract] OR milk[Title/Abstract]) AND (tooth[Title/Abstract] OR teeth[Title/Abstract] OR dental[Title/Abstract] OR dentition[Title/Abstract])))		414
**10**	((dental materials[MeSH Terms]) AND (primary teeth[MeSH Terms])) OR (deciduous dentition[MeSH Terms])		12,496
**11**	(((dental materials[MeSH Terms]) AND (primary teeth[MeSH Terms])) OR (deciduous dentition[MeSH Terms])) AND (((restorati*[Title/Abstract] OR crown[Title/Abstract] OR filling*[Title/Abstract]) AND (cari*[Title/Abstract] OR decay[Title/Abstract] OR cavities[Title/Abstract] OR dentine[Title/Abstract] OR lesion[Title/Abstract]))		881
**12**	(((dental materials[MeSH Terms]) AND (primary teeth[MeSH Terms])) OR (deciduous dentition[MeSH Terms])) AND (((restorati*[Title/Abstract] OR crown[Title/Abstract] OR filling*[Title/Abstract]) AND (cari*[Title/Abstract] OR decay[Title/Abstract] OR cavities[Title/Abstract] OR dentine[Title/Abstract] OR lesion[Title/Abstract]))	Humans	867
**13**	(((dental materials[MeSH Terms]) AND (primary teeth[MeSH Terms])) OR (deciduous dentition[MeSH Terms])) AND (((restorati*[Title/Abstract] OR crown[Title/Abstract] OR filling*[Title/Abstract]) AND (cari*[Title/Abstract] OR decay[Title/Abstract] OR cavities[Title/Abstract] OR dentine[Title/Abstract] OR lesion[Title/Abstract]))	Randomized Controlled Trial, Humans	154
**14**	(((dental materials[MeSH Terms]) AND (primary teeth[MeSH Terms])) OR (deciduous dentition[MeSH Terms])) AND (((restorati*[Title/Abstract] OR crown[Title/Abstract] OR filling*[Title/Abstract]) AND (cari*[Title/Abstract] OR decay[Title/Abstract] OR cavities[Title/Abstract] OR dentine[Title/Abstract] OR lesion[Title/Abstract]))	Randomized Controlled Trial	155
**15**	("glass"[Title/Abstract] OR "polyalkenoate"[Title/Abstract] OR "ionomer"[Title/Abstract] OR "cement*"[Title/Abstract] OR "resin*"[Title/Abstract] OR "metal"[Title/Abstract] OR "composite*"[Title/Abstract] OR "amalgam"[Title/Abstract] OR "compomer*"[Title/Abstract] OR "Polyacid"[Title/Abstract] OR "biomaterial"[Title/Abstract] OR "bio-active"[Title/Abstract]) AND (("restorati*"[Title/Abstract] OR "crown"[Title/Abstract] OR "filling*"[Title/Abstract]) AND ("cari*"[Title/Abstract] OR "decay"[Title/Abstract] OR "cavities"[Title/Abstract] OR "dentine"[Title/Abstract] OR "lesion"[Title/Abstract]) AND (("primary"[Title/Abstract] OR "baby"[Title/Abstract] OR "deciduous"[Title/Abstract] OR "milk"[Title/Abstract]) AND ("tooth"[Title/Abstract] OR "teeth"[Title/Abstract] OR "dental"[Title/Abstract] OR "dentition"[Title/Abstract]))) AND ("control*"[All Fields] OR "random*"[All Fields])		410
**16**	(("dental materials"[MeSH Terms] AND "tooth, deciduous"[MeSH Terms]) OR "tooth, deciduous"[MeSH Terms]) AND (("restorati*"[Title/Abstract] OR "crown"[Title/Abstract] OR "filling*"[Title/Abstract]) AND ("cari*"[Title/Abstract] OR "decay"[Title/Abstract] OR "cavities"[Title/Abstract] OR "dentine"[Title/Abstract] OR "lesion"[Title/Abstract])) AND ("control*"[All Fields] OR "random*"[All Fields])		410

Embase via Ovid search, 28.12.2020		
1	((glass or polyalkenoate or ionomer or cement* or resin* or metal or composite* or amalgam or compomer* or Polyacid or biomaterial or bio-active) and (primary or milk or baby or deciduous)).ab.	71166
2	(caries or decay or cavities or lesion).ab.	688715
3	1 and 2	5570
4	(restorative or restoration or crown or filling).ab.	269167
5	3 and 4	720

Cochrane Library search, 28.12.2020

#1 (glass or polyalkenoate or ionomer or cement* or resin* or metal or composite* or amalgam or compomer* or Polyacid or biomaterial or bio-active):ti,ab,kw (Word variations have been searched) 38813

#2 (glass or polyalkenoate or ionomer or cement* or resin* or metal or composite* or amalgam or compomer* or Polyacid or biomaterial or bio-active):ti,ab,kw AND (primary OR milk OR baby OR deciduous):ti,ab,kw (Word variations have been searched) 18645

#3 (glass or polyalkenoate or ionomer or cement* or resin* or metal or composite* or amalgam or compomer* or Polyacid or biomaterial or bio-active):ti,ab,kw AND (primary OR milk OR baby OR deciduous):ti,ab,kw AND (tooth OR teeth OR molar OR dental):ti,ab,kw (Word variations have been searched) 1677

#4 (glass or polyalkenoate or ionomer or cement* or resin* or metal or composite* or amalgam or compomer* or Polyacid or biomaterial or bio-active):ti,ab,kw AND (primary OR milk OR baby OR deciduous):ti,ab,kw AND (tooth OR teeth OR molar OR dental):ti,ab,kw AND (caries OR decay OR cavities):ti,ab,kw (Word variations have been searched) 944

LILACS search, 28.12.2020

primary OR milk OR baby OR deciduous [Abstract words] and restoration OR restorative OR crown OR filling [Words]
484

primary OR milk OR baby OR deciduous [Abstract words] and restoration OR restorative OR crown OR filling [Abstract words]
395

primary OR milk OR baby OR deciduous [Abstract words] and glass OR polyalkenoate OR ionomer OR cement* OR resin* OR metal OR composite* OR amalgam OR compomer* OR Polyacid OR biomaterial OR bio-active [Words]
0

primary OR milk OR baby OR deciduous [Abstract words] and restoration OR restorative OR crown OR filling [Abstract words] and caries OR cavities OR decay [Abstract words]
107

primary OR milk OR baby OR deciduous [Abstract words] and restoration OR restorative OR crown OR filling [Abstract words] and dental materials [Words]
8
